# MicroRNA-195 acts as an anti-proliferative miRNA in human melanoma cells by targeting Prohibitin 1

**DOI:** 10.1186/s12885-017-3721-7

**Published:** 2017-11-10

**Authors:** Priscila Daniele Ramos Cirilo, Luciana Nogueira de Sousa Andrade, Bruna Renata Silva Corrêa, Mei Qiao, Tatiane Katsue Furuya, Roger Chammas, Luiz Otavio Ferraz Penalva

**Affiliations:** 1 Instituto do Câncer do Estado de São Paulo, Centro de Investigação Translacional em Oncologia, Laboratório de Oncologia Experimental, Av. Dr. Arnaldo,251, São Paulo, SP CEP 01246-000 Brazil; 2 The University of Texas Health Science Center at San Antonio, Children’s Cancer Research Institute, 7703 Floyd Curl Drive, San Antonio, TX 78229-390 USA; 3 Instituto Hermes Pardini, Setor de Pesquisa e Desenvolvimento, Av das Nações, 2448, Distrito Industrial, Vespasiano, MG CEP 33200-000 Brazil; 4 Instituto Sírio-Libanês de Ensino e Pesquisa, Centro de Oncologia Molecular, Rua Prof. Daher Cutait, 69, São Paulo, SP CEP 01308-060 Brazil

**Keywords:** Melanoma, microRNA-195, Prohibitin 1, Cisplatin, Temozolomide, Vemurafenib

## Abstract

**Background:**

Melanoma is the most lethal type of skin cancer. Since chemoresistance is a significant barrier, identification of regulators affecting chemosensitivity is necessary in order to create new forms of intervention. Prohibitin 1 (PHB1) can act as anti-apoptotic or tumor suppressor molecule, depending on its subcellular localization. Our recent data shown that accumulation of PHB1 protects melanoma cells from chemotherapy-induced cell death. Lacking of post-transcriptional regulation of PHB1 could explain this accumulation. Interestingly, most of melanoma patients have down-regulation of microRNA-195. Here, we investigate the role of miR-195, its impact on PHB1 expression, and on chemosensitivity in melanoma cells.

**Methods:**

TCGA-RNAseq data obtained from 341 melanoma patient samples as well as a panel of melanoma cell lines were used in an expression correlation analysis between *PHB1* and predicted miRNAs. miR-195 impact on PHB1 mRNA and protein levels and relevance of this regulation were investigated in UACC-62 and SK-MEL-5 melanoma lines by RT-qPCR and western blot, luciferase reporter and genetic rescue experiments. Cell proliferation, cell-cycle analysis and caspase 3/7 assay were performed to investigate the potential action of miR-195 as chemosensitizer in melanoma cells treated with cisplatin and temozolomide.

**Results:**

Analysis of the TCGA-RNAseq revealed a significant negative correlation (Pearson) between miR-195 and PHB1 expression. Moreover, RT-qPCR data showed that miR-195 is down-regulated while PHB1 is up-regulated in a collection of melanoma cells. We demonstrated that miR-195 regulates PHB1 directly by RT-qPCR and western blot in melanoma cells and luciferase assays. To establish PHB1 as a relevant target of miR-195, we conducted rescue experiments in which we showed that PHB1 transgenic expression could antagonize the suppressive effect miR-195 on the proliferation of melanoma cells. Finally, transfection experiments combined with drug treatments performed in the UACC-62 and SK-MEL-5 melanoma cells corroborated miR-195 as potential anti-proliferative agent, with potential impact in sensitization of melanoma cell death.

**Conclusions:**

This study support the role of miR-195 as anti-proliferative miRNA via targeting of PHB1 in melanoma cells.

**Electronic supplementary material:**

The online version of this article (10.1186/s12885-017-3721-7) contains supplementary material, which is available to authorized users.

## Background

Melanoma is the most aggressive and lethal type of skin cancer. It has been reported to be the fifth and seventh most common cancer type in the US among men and women, respectively [1]. The National Cancer Institute estimates that 76,380 new cases of melanoma were diagnosed and about 10,000 people have died from this disease in the US in 2016. Most melanomas diagnosed at stage 0-III are excised surgically, with lymph node management. However, unresectable stage III, IV and recurrent melanomas are treated with chemotherapy, targeted therapy or immunotherapy [1].

Cutaneous melanoma is classified into four subtypes based on the status of the most significant mutated genes: *BRAF*, *RAS*, *NF1*, and Triple-WT (wild-type) [2]. About 50% of patients harboring a BRAF V600E mutation show good response rates (about 80%) after receiving targeted therapies such as vemurafenib (PLX-4032), but the average duration of disease-free survival is less than six months [3]. Immunotherapy has been used to treat metastatic melanoma with significant improvement in overall survival and progression-free survival compared to chemotherapy [4]. Therapeutic strategies using conventional chemotherapy, alone or in combination with other therapies, are under investigation to improve the efficacy of treatment of metastatic melanoma [5, 6]. Better knowledge of the molecular mechanisms and signaling pathways associated with chemoresistance in melanoma is necessary to design novel therapeutic strategies.

Melanoma arises from malignant transformation of melanocytes induced mainly by exposure to intense intermittent ultraviolet radiation, an optimal oxidative stress microenvironment [7]. Thus, melanoma cells originate under stress conditions, which favor their therapy-resistant phenotype. Proteomic assays performed in our laboratory have shown that melanoma cells exposed to high doses of cisplatin (25 μM) induced accumulation of anti-apoptotic molecules and proteins involved in the oxidative stress response, including Prohibitin 1 [8]. The human Prohibitin 1 gene (*PHB1*) is located on chromosome 17q21 and encodes PHB1, a highly conserved protein that is ubiquitously expressed in many cell types [9]. A growing body of evidence indicates that the subcellular localization of PHB1 is a determinant of its function [10–12]. At the level of the cell plasma membrane, PHB1 is a transmembrane adaptor that activates downstream signal transduction. It has been reported that C-RAF stabilization in the RAS-RAF-MEK-ERK pathway depends on PHB1 [13]. PHB1 may serve as a novel druggable target in C-RAF-mediated vemurafenib resistance since treatment with the natural compound rocaglamide A disrupts the interaction between PHB1 and C-RAF in melanoma cells [14]. In the nucleus, PHB1 regulates transcriptional activation, cell cycle and E2F function [15]. In the mitochondrial inner membrane, PHB1 and PHB2 heterodimers are implicated in mitochondrial genome stabilization, mitochondrial morphology, oxidative stress, and apoptosis [9, 16]. We observed PHB1 accumulation in the mitochondria and nucleus of melanoma cells after high doses of cisplatin and demonstrated that PHB1 knockdown sensitizes melanoma cells to cisplatin-induced cell death [8].

MicroRNAs (miRNAs) are important regulators of gene expression, functioning via translation repression and/or mRNA degradation (for review see [17]). Aberrantly expressed miRNAs have been shown to initiate or drive the progression of cancer, acting as potential oncogenes or tumor suppressors in several tumor types, including melanoma [18, 19]. There is a growing body of evidence that the involvement of miRNAs is crucial in the progression of metastatic melanoma. Down-regulation of miR-137 in melanoma was strongly associated with *MITF* up-regulation, one of the most important gene involved with melanoma risk (for review see [20]). MicroRNA-7, for example, is downregulated in VemR A375 and Mel-CV melanoma cells, both resistant to vemurafenib. Reestablishment of miR-7 expression reverse this resistance by targeting EGFR/IGF-1R/CRAF pathway [21]. Recently, Li et al. [22] showed that microRNA-488-3p sensitizes malignant melanoma cells to cisplatin by targeting *PRKDC* gene. Therefore, lacking of post-transcriptional mechanisms involved in drug resistance such as intrinsic tumor down-regulation of miRNAs could induce up-regulation of chemoresistance-related genes [23]. Here, we demonstrate that miR-195, a classical tumor suppressor in many types of cancer, is down-regulated in melanoma and directly regulates PHB1 expression. Moreover, miR-195 mimics impact cancer related phenotypes and modulate drug response in melanoma cells.

## Methods

### Analysis of melanoma samples from the Cancer Genome Atlas

The miRanda Database was used to generate a list of miRNAs predicted to target *PHB1*. Data from The Cancer Genome Atlas (TCGA) were used to evaluate the expression of miR-195 and *PHB1*. We downloaded level 3 data of 341 matched mRNA-Seq and miRNA-Seq tumor samples, as well as one normal sample for each data set. Pearson correlation was used to calculate pairwise correlations between *PHB1* and miRNAs expression. Gene expression analyses comparing melanoma samples with normal samples were performed using EdgeR [24].

### Cell lines

Human melanoma cell lines SK-MEL-5, SK-MEL-19, SK-MEL-37, SK-MEL-147, UACC-62, WM35, WM793B, WM1366, WM1552C, WM1617, Lox10, MZ2Mel, and Human immortalized keratinocytes (HaCat) were maintained with DMEM (Gibco/Thermo Fisher Scientific, Waltham, MA, USA) medium supplemented with 10% fetal bovine serum (FBS) and antibiotics (10,000 units/mL of penicillin and 10,000 μg/mL of streptomycin). Human melanocytes (NGM) were maintained with DMEM/F-12 medium supplemented with 20% FBS and 1% Human Melanocyte Growth Supplement (HMGS) (LifeTechnologies/Thermo Fisher Scientific, Waltham, MA, USA). HeLa cells were maintained with RPMI medium supplemented with 10% FBS and antibiotics. The sources of all cell lines used at this study are described in detail in Additional file 1: Table S1. UACC-62 and SK-MEL-5 were selected for functional assays since these lines were isolated from metastatic melanoma and are positive for the BRAF-V600E mutation [25]. Cells were screened monthly for *Mycoplasma* contamination.

### MicroRNAs mimics transfection

UACC-62 and SK-MEL-5 cells were transfected with microRNA mimics using Lipofectamine RNAiMAX transfection reagent (Invitrogen/Thermo Fisher Scientific, Waltham, MA, USA). We used miRNA mimic Syn-has-miR-195 (5′-TCCTTCATTCCACCGGAGTCTG-3′) (GE Dharmacon, Lafayette, CO USA) and ALL STARS Negative control siRNA (QIAGEN, Hilden, Germany). PHB1 expression in melanoma cells was evaluated by quantitative real time polymerase chain reaction (RT-qPCR) and western blot 48 h (24 h mimics plus 24 h of drugs) and 72 h (24 h mimics plus 48 h of drugs) after treatment, respectively.

### siRNAs transfection

Stable UACC-62 cells expressing PHB1 were reversely transfected with four siRNAs (25 nM) sequences targeting PHB1 (Dharmacon, ON-TARGETplus SMARTpool siRNA J-010530-05,-06,-07, and −08, Thermo Scientific) using Lipofectamine RNAiMAX transfection reagent (Invitrogen/Thermo Fisher Scientific, Waltham, MA, USA). Negative control ON-TARGETplus Non-targeting siRNA reagent (D-001810-01-05) was obtained from Dharmacon. Endogenous and recombinant PHB1 expression were evaluated 72 h after siRNA transfections and identified by immunoblotting assay.

### Plasmids construction and site-directed mutation

A 852 bp (position 82–934) fragment of PHB1 3’UTR region (PHB1–3’UTR-WT) was synthesized by GeneArt System (Invitrogen/Thermo Fisher Scientific, Waltham, MA, USA) and sub-cloned into the pmirGLO Dual-Luciferase miRNA Target Expression vector (Promega, Madison, WI USA) at NheI/XhoI restriction sites. Site-directed mutation was performed in order to delete miR-195 binding-site region (PHB1–3’UTR-del195–5′-…agaTGCTGCTgaa…3′) using *Pfu* Turbo DNA polymerase (2.5 U/μL) following the manufacturer’s instructions (Stratagene, La Jolla, CA, USA). PHB1-ORF (819 bp) was cloned into a pENTR223 cassette in an ORFExpress System (GeneCopoeia, Rockville, MD USA) and then into a pcDNA3.1-nV5-DEST plasmid using the Gateway System (Invitrogen/Thermo Fisher Scientific, Waltham, MA, USA). Sanger sequencing confirmed all construct inserts.

### Stable cell lines generation

UACC-62 cells stably expressing PHB1-ORF (Open Reading Frame, without 5′ and 3’UTR) or pcDNA3.1-EV (empty vector) (Invitrogen/Thermo Fisher Scientific, Waltham, MA, USA) were generated by transfection followed by G418 selection (Gibco/Thermo Fisher Scientific, Waltham, MA, USA) (0.8 mg/mL). Plasmid transfections were carried out using the Lipofectamine 3000 reagent (Invitrogen/Thermo Fisher Scientific, Waltham, MA, USA). The PHB1 expression level was monitored using immunoblotting assays.

### Quantitative RT-PCR

After lysis with TRIzol® reagent (Invitrogen/Thermo Fisher Scientific, Waltham, MA, USA), total RNA was isolated from the aqueous phase upon mixing with chloroform, precipitated with isopropanol, washed with 75% ethanol and re-suspended in nuclease-free water. cDNA was synthesized using the High Capacity cDNA Reverse Transcription Kit (Applied Biosystems/Thermo Fisher Scientific, Waltham, MA, USA). Quantitative RT-PCR for *PHB1* (Fwd: 5′-GTGTGGTTGGGGAATTCATGTGG-3′; Rev.: 5′-CAGGCCAAACTTGCCAATGGAC-3′), and endogenous control *A-CTB* (Fwd: 5′-CCTGGCACCCAGCACAAT-3′; Rev.: 5′-GGGCCGGACTCGTCATACT-3′) were carried out using SYBR Green Master Mix (Applied Biosystems/Thermo Fisher Scientific, Waltham, MA USA). The miRNA-195 or RNU48 (endogenous control) transcripts were quantified using TaqMan Small RNA assays (Applied Biosystems/Thermo Fisher Scientific, Waltham, MA, USA). All reactions were performed in an ABI 7500 Real Time PCR machine (Applied Biosystems/Thermo Fisher Scientific, Waltham, MA USA) and data were acquired using the ABI SDS 2.0.1 software package and analyzed using the 2^-^^∆∆Ct^ method.

### Immunoblotting

After collection, cells were suspended and sonicated in 2xSDS Laemmli sample buffer. A 12% SDS-PAGE gel with a 4% stacking gel was run in Tris-glycine- SDS buffer. A semi-dry transfer procedure onto a nitrocellulose membrane was carried out. After transfer, the membrane was blocked with Tris-buffered saline (TBS) with 1% Tween-20 and 5% milk. Membranes were probed with a goat polyclonal anti-Prohibitin 1 antibody (PHB1, 1:200, Santa Cruz, Santa Cruz, CA USA), mouse-V5 Tag Monoclonal antibody (V5-Tag, 1:4000, Invitrogen), mouse monoclonal anti-alpha Tubulin antibody (TUB, 1:2000, Sigma), and monoclonal anti-beta-actin antibody (ACT-B, 1:2000, Abcam, Cambridge, UK). Horseradish peroxidase (HRP)-conjugated anti-Goat IgG antibody (1:6000) was used as a secondary antibody for anti-PHB1 while HRP-conjugated goat anti-mouse IgG antibody (Pierce) was used as a secondary antibody for anti-TUB and anti-ACT-B (for both 1:4000) and for anti-V5-Tag (1:8000). Proteins were detected using the electro-chemoluminescence FluorChem R System (Protein Simple, San Jose, CA, USA).

### Cell proliferation assay

Cell proliferation assay was conducted using UACC-62 and SK-MEL-5 cell lines seeded in 96-well plates (3 × 10^3^ cells per well). Cells were reverse transfected (RNAiMax) with miRNA-195/miRNA-control mimics (10 nM). After 24 h, cells were treated with cisplatin (2.5, 5.0 and 10 μM, SIGMA, Darmstadt, Germany), temozolomide (50, 250 and 450 μM, SIGMA, Darmstadt, Germany) or DMSO 0.1% as vehicle. Forty-eight hours after treatment, the nuclear counting per mm^2^ (%) of treated cells was compared to the non-treated cells (IncuCyte, Essen BioScience, Ann Arbor, MI, USA). For miR-195-PHB1 antagonism studies, two clones of the UACC-62 cell line overexpressing either ORF-PHB1 or pcDNA3.1-EV were used. MicroRNA-195 or miRNA-control was transfected into stable each cell line. Nuclear counting per mm^2^ was carried out daily for five days after transfection using IncuCyte software and viability of control (%) was calculated.

### Cell death and cell cycle analysis

UACC-62 and SK-MEL-5 cells were seeded at 2 × 10^5^ cells per well in a 12 multiwell plate. Cells were reverse transfected (RNAiMax) with miRNA-195/miRNA-control mimics (10 nM). After 24 h, cells were treated with cisplatin (2.5 and 10 μM), temozolomide (50 and 250 μM) or DMSO 0.1%. After 48 h, cells were trypsinized, fixed in 70% ethanol and kept at −20 °C until analysis by flow cytometry (Attune® Acoustic Focusing Cytometer, Applied Biosystems/Thermo Fisher Scientific, Waltham, MA, USA). Cell death and cell cycle analysis were performed by propidium iodide (PI) staining. PI incorporates stoichiometrically to DNA, allowing relative quantitation of DNA content. Cell death analysis, indicated as hypodiploid cells (Sub-G1) and cell cycle distribution (G0/G1, S, and G2/M) analysis were performed using the FlowJo v10 Cytometric Software algorithm (FlowJo LLC, Ashland, Oregon, USA). The percentage of cell death was expressed in bar graphs (GraphPad, La Jolla, CA). Cell cycle distribution profiles were plotted in a chart.

### Caspase 3/7 apoptosis assay

A caspase 3/7 activity-based assay was performed for apoptosis quantification. UACC-62 and SK-MEL-5 cells were seeded in 96 well plates and reverse transfected with either miR-195 or miRNA-control (10 nM). After 24 h, cells were exposed to cisplatin or temozolomide (2.5 and 50 μM, respectively). After 48 h, the apoptosis index was monitored in the supernatant using the Caspase-Glo 3/7 Assay Reagent according to manufacturer’s instructions (Promega, Madison, WI, USA). Luciferase measurements were performed with the SpectraMax M5 Multi-Mode Microplate Reader (Molecular Devices, Sunnyvale, CA, USA).

### Dual-GLO luciferase assay

For the luciferase assays, 8 × 10^3^ HeLa cells were plated 24 h prior to plasmid transfection in a 96-well plate in triplicate. 10 ng of each pmiR-GLO-3′-UTR-PHB1 or pmiR-GLO-PHB1–3’UTR-del195 reporter vector were mixed with 500 nM of each miRNA-195 or miRNA-control in 25 μL OptiMEM (Invitrogen/Thermo Fisher Scientific, Waltham, MA USA). A 0.5 μL aliquot of Lipofectamine 2000 transfection reagent (Invitrogen/Thermo Fisher Scientific, Waltham, MA USA) was added in 25 μL OptiMEM. Mixes were combined and after formation of the nucleic acid:lipid complex, the transfection solution was overlaid onto the previously plated HeLa cells. HeLa cells were selected for luciferase assays based on their high transfection efficiency and reproducibility according to our previous experience [26, 27]. After incubation for 48 h, a HeLa cell extract was prepared using the Reporter Lysis Buffer (Promega, Madison, WI, USA). A 50 μL amount of Luciferase Assay Reagent (Promega, Madison, WI USA) was added to 10 μL of cell lysate and luminescence was measured with a GloMax-Multi + Microplate Multimode Reader (Promega, Madison, WI, USA). Data were normalized by Firefly/Renilla luciferase activity.

### Statistical analysis

Statistical analyses were conducted using GraphPad Prism Software v6.01 (GraphPad, La Jolla, CA). The difference between two groups were analyzed by the unpaired *t* test. The differences between three or more groups were analyzed by ANOVA with Tukey’s multiple comparisons test. A value of *P* ≤ 0.05 was considered to be statistically significant.

## Results

### PHB1 expression is negatively correlated with miRNA-195 expression

**Fig. 1:**
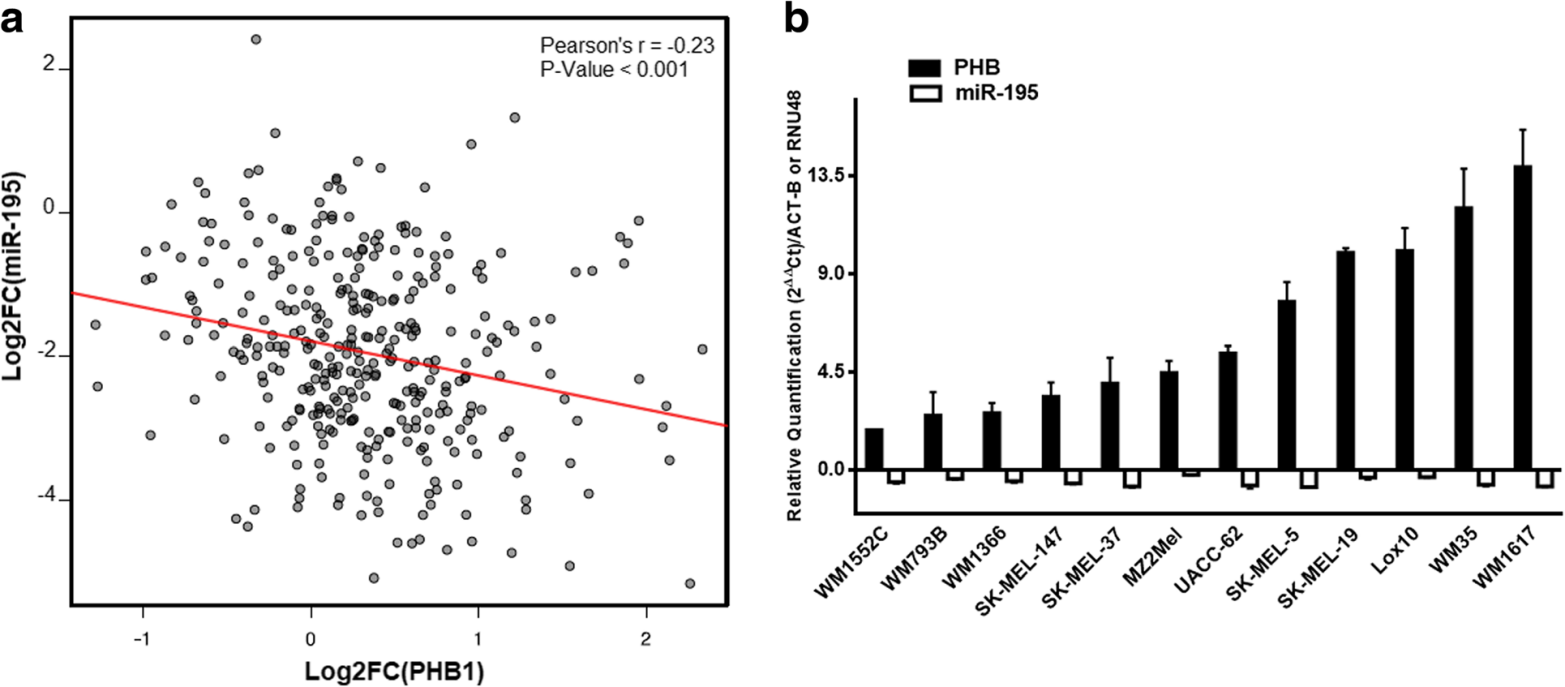
MicroRNA-195 is down-regulated and *PHB1* is up-regulated in patient and melanoma cell lines. **a** Scatter plot of the RNA Sequence data (TCGA) of 341 samples from melanoma patients compared to normal skin samples. The red line indicates an inverse correlation of expression between the samples for miR-195 and *PHB1* genes (Pearson’s *r* = −0.23; *P* ≤ 0.001). **b** MicroRNA-195 is down-regulated (open columns down) and *PHB1* up-regulated (full columns up) in 12/12 melanoma cell lines evaluated by RT-qPCR compared to melanocytes (NGM cells) using 2^(− ∆∆ Ct)^ method. TCGA data are reported as means ± SD of relative quantification Log_2_ base values

To define regulators that could influence the expression of PHB1 in melanoma, we looked into the miRanda Database and identified 28 miRNAs with putative sites in the *PHB1* 3’UTR region. Next, we conducted an expression correlation analysis. We identified 341 melanoma samples (melanoma skin cancer type) in The Cancer Genome Atlas and examined mRNA-Seq and miRNA-Seq data. We checked *PHB1* and miRNAs expression levels in control and tumor samples to determine which miRNAs from the candidate list showed the strongest anti-correlation with *PHB1*. We calculated Pearson correlations between the fold-changes of *PHB1* and each of the miRNAs. Among the top three negatively correlated miRNAs, miRNA-195 caught our attention (Pearson’s *r* = −0.23; *P* < 0.001, Fig. 1a). miR-195 acts as a classical tumor suppressor miRNA in many tumor types and regulates anti-apoptotic molecules in drug resistance pathways [28]. To corroborate the observed negative correlation, we analyzed the gene expression levels of *PHB1* and miR-195 in 12 melanoma cell lines compared to melanocytes (NGM) (Fig. 1b). Taken together, these data indicate that *PHB1* up-regulation in melanoma could be due in part to a decrease in miR-195 expression.

### PHB1 expression is modulated by miRNA-195

**Fig. 2:**
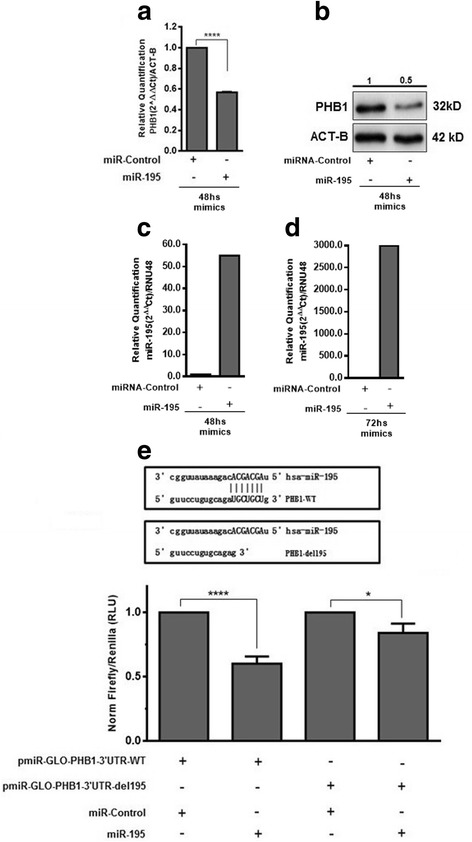
MicroRNA-195 modulates PHB1 expression in melanoma cells and in a gene reporter assay. UACC-62 melanoma cells were transfected with either miR-control/mir-195. miR-195 mimics transfection produced a significant reduction of PHB1 (*P* ≤ 0.0001) (**a**) mRNA and (**b**) protein levels compared to miR-control. For RT-qPCR experiments, *ACT-B* mRNA was used as an endogenous control and the data were analyzed using the 2 ^(−∆∆Ct)^ method; for immunoblotting ACT-B was also used as loading control. Protein quantification (fold-change based on the control) is indicated above the blots. In (**c**) and (**d**) miR-195 levels 48 and 72 h after transfection, respectively. RNU48 was used as an endogenous control and the data were analyzed using the 2 ^(−∆∆Ct)^ method. (**e**) Schematic representation of the PHB1–3’UTR region. pmiR-GLO-PHB1–3’UTR wild type (PHB1-WT) was submitted to a mutagenesis assay to delete the miR-195 binding-site sequence (PHB1-del195). HeLa cells were transiently co-transfected with either pmiR-GLO-PHB1–3’UTR-WT/pmiR-GLO-PHB1–3’UTR-del195 in the presence of miRNA-control/miR-195 mimics. After 48 h, Firefly and Renilla luciferase activity was measured and normalized. Results shown that miR-195 decreased luciferase activity by about 40% (*P* ≤ 0.0001). Statistical analysis was carried out using the unpaired *t* test and data are reported as means ± SD. Representative examples of at least three independent experiments are reported. *P ≤ 0.05; ****P ≤ 0.0001

To investigate whether miR-195 regulates directly PHB1 expression, UACC-62 and SK-MEL-5 melanoma cells were transfected with miR-195 mimics or a miR-control. After 24 and 48 h, cells were collected for mRNA and protein quantification, respectively. PHB1 mRNA decreased by approximately 50% in UACC-62 and by 20% in SK-MEL-5 cells upon miR-195 transfection (Fig. 2a and Additional file 2: Figure S1A, *P* ≤ 0.0001 and *P* ≤ 0.01, respectively). PHB1 protein levels were decreased by approximately 50% and 30% in UACC-62 and SK-MEL-5 cells after miR-195 mimics transfection compared to miR-control (Fig. 2b and Additional file 2: Figure S1B, respectively). In addition, miR-195 is still up-regulated even 48 and 72hs after transfection (Fig. 2c and d and Additional file 2: Figure S1C and D, respectively. To confirm that PHB1 is a direct target of miR-195, an 852 bp fragment of the 3′-UTR of PHB1 containing the putative miR-195 binding site was cloned (pmiR-GLO-PHB1–3’UTR-WT) and a miR-195 binding site deletion clone was prepared (pmiR-GLO-PHB1–3’UTR-del195) (Fig. 2e, upper panel). A co-transfection experiment showed that miR-195 decreased the expression of pmiR-GLO-PHB-3’UTR-WT by approximately 40%, based on luciferase/renilla activity (*P* ≤ 0.0001). Deletion of miR-195 binding site in PHB1 3′ UTR decreased the regulation (*P* ≤ 0.05) (Fig. 2e, lower panel). In fact, the deletion of miR-195 binding site in PHB 3′ UTR reduced drastically the effect of miR-195 but did not completely abolished it. We checked the sequence of PHB 3’UTR and identified another sequence that partially matches miR-195 seed sequence. It is possible that this site is weakly recognized by miR-195 and contributes to the regulation.

### PHB1 antagonizes the suppressive effect of miRNA-195 on cell proliferation

**Fig. 3:**
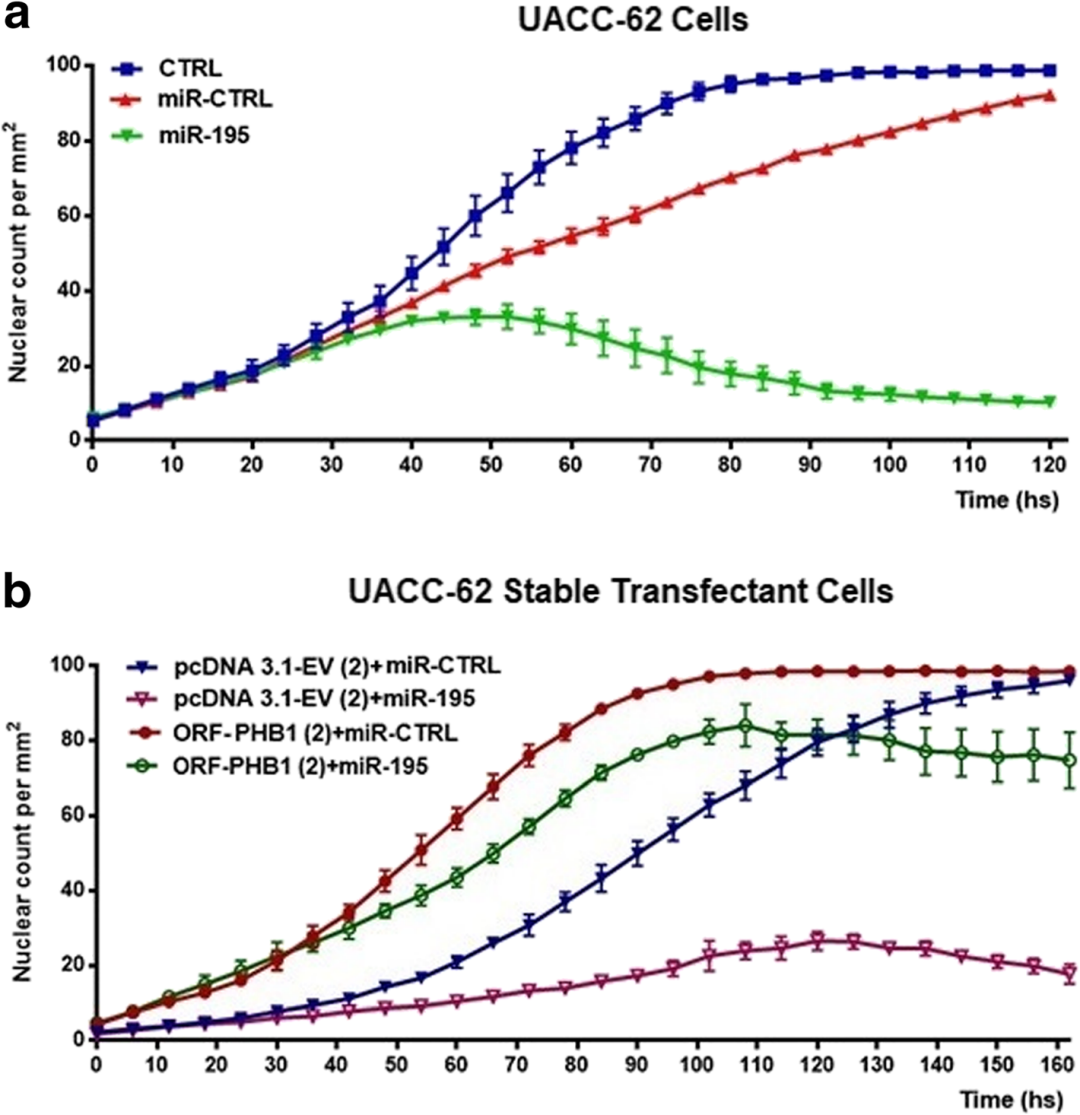
PHB1 overcomes the anti-proliferative effect of miRNA-195. (**a**) Proliferation assay based on nuclear counting per mm^2^. UACC-62 melanoma cells were transfected with either miR-control or miR-195 (25 nM) and observed for five days after transfection. (**b**) To conduct rescue experiments, UACC-62 melanoma cells were stably expressing either ORF-PHB1 or pcDNA3.1-EV. Cells were then transfected with either miRNA-mimics control or miR-195 mimics. After transfection, the proliferation rate was measured for six days and the results showed that cells transfected with transgenic PHB1 overcome the suppressive effect of miR-195 (green line) compared to pcDNA3.1-EV cells (pink line). Representative examples of at least three independent experiments are reported

To determine the anti-proliferative effect of miR-195, UACC-62 melanoma cells were transfected with either miR-195 or miR-CTRL mimics (10 nM) and the proliferative indices were plotted as a survival curve for five days. Figure 3a shows the proliferation curve for cells transfected with miR-CTRL, reaching a 90% proliferation rate at 120 h, while cells with miR-195 reached a 10% proliferation rate at the same time point. Similar results were observed for SK-MEL-5 (Additional file 3: Figure S2). To determine if this suppressive effect of miR-195 takes place primarily via PHB1 inhibition, we conducted rescue experiments. UACC-62 stable cells containing a PHB1 open reading frame construct or pcDNA3.1 empty vector (EV) were generated and transfected with mimics under the same conditions as described above. The stable expression of transgenic PHB1 was confirmed by immunoblotting (Additional file 4: Figure S3). The proliferative index was plotted for 6.5 days (Fig. 3b). UACC-62-EV cells and cells transfected with miR-CTRL reached the saturation density along 120 h and showed a proliferation index of about 100% at the 160 h time-point (Fig. 3b). However, when these cells were transfected with miR-195, the proliferation index decreased to 18–30%. In cells transfected with the open reading frame (ORF) of PHB1, and therefore not susceptible to miR-195 inhibition, a different scenario was observed. When miR-CTRL was transfected in ORF-PHB1 expressing cells, the proliferation index reached its maximum in 99 h (Fig. 3b), while miR-195 mimics transfection produced a much less dramatic impact on the proliferation index, which reached the maximum of 80% in 100 h (Fig. 3b). These results indicate that the anti-proliferative effect of miR-195 observed in melanoma cells was in great part due to PHB1 regulation.

### Effect of miRNA-195 and drugs in melanoma cells

**Fig. 4:**
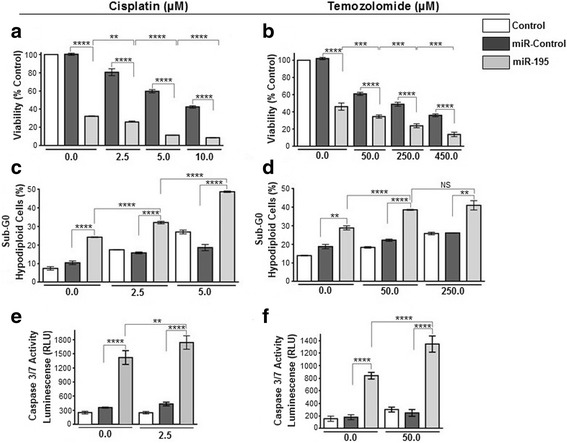
MicroRNA-195 and drugs effect in UACC-62 melanoma cells. (**a**-**b**) Cell viability rate was calculated based on the proliferation index ratio (%) of treated cells/not treated cells (control). Increasing doses of cisplatin (2.5, 5.0, and 10.0 μM) and temozolomide (50, 250, and 450 μM) were tested. (**c**-**d**) FlowJo Cytometry Analysis software was used for hypodiploid cell quantification after propidium iodide staining. Cells were treated with 2.5 and 5.0 μM cisplatin and 50 and 250 μM temozolomide drugs. (**e**-**f**) Apoptosis index based on caspase 3/7 activity was measured in a luminometer. All results showed that miR-195 exerts a small effect in UACC-62 melanoma cells sensitization to cisplatin and temozolomide treatments. All experimental data were obtained 24 h after miRNA-control/miR-195 (10 nM) transfection plus 48 h of drug exposure (total time 72 h). Statistical analysis was carried out using ANOVA with multiple comparison test and are reported as means ± SD. Representative data of at least three independent experiments are reported. NS: non-significant; **P ≤ 0.01;***P ≤ 0.001; **** *P* ≤ 0.0001

**Fig. 5:**
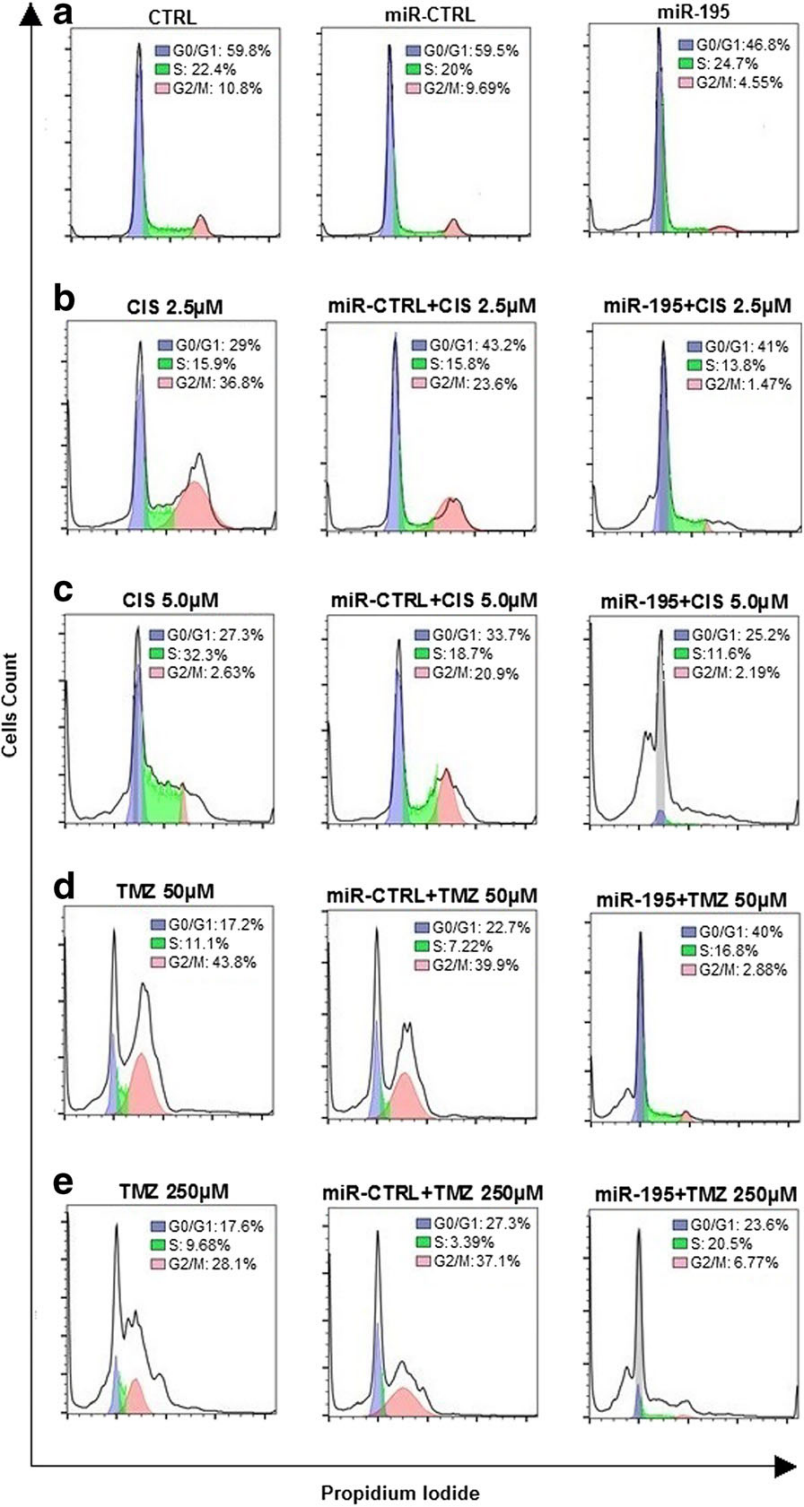
Drug-induced cell death is accentuated by miR-195. This panel shows the cell cycle profile of UACC-62 melanoma cells transfected with either miRNA-control/miR-195 (10 nM) (24 h) and treated with cisplatin (CIS-2.5 and 5 μM) or temozolomide (TMZ-50 and 250 μM) for 48 h (total time 72 h). The percentage of the cell population distributed in each cell cycle phase is indicated: G0/G1 = blue, S = green, and G2/M = pink. (**a**) MicroRNA-195 alone increased cell death (cells accumulated at sub G0/G1). (**b**-**e**) Treatment with drugs induces mainly arrest of UACC-62 cells in G2/M whereas the cytotoxic effects of cisplatin and temozolomide were higher when combined with miR-195 transfection, inducing cell death (sub G0/G1 cells population). Cell cycle distribution of propidium iodide (PI)-labeled cells was analyzed using FlowJo Cytometric software. Representative examples of at least three independent experiments are reported

We tested if miRNA-195 mimics could potentially sensitize melanoma cells to chemotherapy treatments. First, we did a dose-response curve with increasing doses of cisplatin (2.5, 5.0 and 10 μM) and temozolomide (50, 250 and 450 μM) in UACC-62 and SK-MEL-5 melanoma cells to determine the ideal drug dosage to be used in the assays (data not shown). Then, we transfected both cell lines with either miR-control or miR-195 mimics, expose them to cisplatin and temozolomide and checked the impact on cell death. When cells were treated with increasing doses of cisplatin or temozolomide, cell viability decreased in a dose-dependent manner for both cell lines and miR-195 seems to exert an slightly additive effect combined with drugs on cell viability (Fig. 4a, b and Additional file 5: Figure S4A, B, respectively). Percentage of hypodiploid cells was used as an indicator of cell-death (Sub-G1 population). Interestingly, hypodiploid cells were observed after miR-195 transfection in both UACC-62 (25%) and SK-MEL-5 (40%) cells; however, in the presence of either cisplatin and temozolomide, we did observe a significant increase in cell death index in both cell lines transfected with miR-195, suggesting an effect of miR-195 in melanoma sensitivity to chemotherapy (Fig. 4c, d; Additional file 5: Figure S4C, D, respectively). To check if miR-195 also sensitizes melanoma cells to BRAF inhibitor (vemurafenib, PLX4032), we transfected UACC-62 melanoma cells with miR-195 (25 nM) and treated with 1 μM and 10 μM PLX-4032 for 48 h. The results confirmed the sensitizing role of miR-195 also to target therapy against melanoma (Additional file 6: Figure S5). To confirm that miR-195 induces cell death, we quantified caspase 3/7 activation. As observed in Fig. 4e, f and Additional file 5: Figure S4E, F, miR-195 alone is sufficient to trigger apoptosis and when cells were treated with cisplatin or temozolomide, activation of apoptosis was induced. Moreover, both cisplatin and temozolomide caused accumulation of UACC-62 and SK-MEL-5 cells in the G2/M (Fig. 5b, d, Additional file 7: Figure S6B, D, respectively) phase as already described in previous studies [29, 30]. We also observed an S-phase arrest when UACC-62 cells were treated with 5 μM cisplatin (Fig. 5c). Interestingly, in the presence of miR-195, the cytotoxic effects of cisplatin and temozolomide were even higher and, in this scenario, cell death was not preceded by a cell cycle arrest at the G2/M phase (Fig. 5, and Additional file 7: Figure S6).

## Discussion

We investigated the regulation of *PHB1* by miR-195 and its possible impact on chemoresistance of metastatic melanoma cell lines harboring a BRAF-V600E mutation. Prohibitin 1 is a molecule with multiple functions, most of them involving the protection of cells from various stresses [31]. These stresses are associated with mitochondrial dysfunction and can be involved in the etiology of cancers and/or their response to chemotherapy. Fraser et al. (2003) described a hypothetical model of chemoresistance in human ovarian cancer cells resistant to cisplatin in which PHB1 accumulation in mitochondria impaired pro-caspase 9 activation and apoptosis was suppressed [12]. Indeed, recent results from our laboratory have shown that PHB1 accumulates in mitochondria after stress induced by cisplatin in melanoma cells [8]. Besides that, melanoma cells stably expressing PHB1 were resistant to treatment with cisplatin and temozolomide (Additional file 8: Figure S7). These results indicated that increased expression of PHB1 in this context could be part of a protective response of cells, which in turn could protect cells against cell death. PHB1 is regulated by multiple post-translational modifications and its phosphorylation induces PI3K⁄Akt and RAF⁄ERK pathways, as well as TGF-b cell signaling in cancer cells (for review see [32]). In addition, pharmacological inhibition of PHB1/C-RAF by rocaglamides A (RocA) inhibits RAS-ERK activation and blocks in vitro and in vivo growth and metastasis of pancreatic and melanoma cells [33]. However, the mechanisms of post-transcriptional regulation of PHB1 are not completely understood.

Since the *PHB1* transcript has an extremely long and highly conserved 3’UTR, the case for regulation at the post-transcriptional level is persuasive. Furthermore, the presence of Single Nucleotide Polymorphisms (SNPs) in the PHB1–3’UTR (SNP rs6917) region has been associated with an increased risk of breast cancer and melanoma, whereas the rare allele of this SNP was associated with reduced PHB1 mRNA levels in gastric cancer [34–36]. These SNPs could modulate the binding site of regulatory elements such as microRNAs and regulate transcript decay [37].

MicroRNA-195 is down-regulated in melanoma cells according to the TCGA database and shows a significant negative expression correlation with PHB1. It is also down-regulated in all melanoma cell lines we tested with respect to melanocytes. miR-195 is located at 17p13.1 and belongs to the microRNA-15/16/195/424/497 family [38]. miR-195 is described as a classical tumor suppressor in many tumors and down-regulation of the miR-195/497 cluster could be explained by a hypermethylated promoter region in hepatocellular carcinoma, breast cancer, gastric cancer, and glioblastoma [39–42]. Transfection of miRNA-195 mimics down-regulates PHB1 mRNA and protein levels in UACC-62 and SK-MEL-5. miR-195 has a slightly sensitize effect in human melanoma cells to different doses of cisplatin and temozolomide. This was observed with the occurrence of a decrease in cell viability and an increase in hypodiploid cells and caspase 3/7 activation. Previous studies have shown that ectopic expression of miR-195 also sensitized glioblastoma, hepatocellular carcinoma, breast cancer, and colon tumor cells to temozolomide, 5-fluorouracil, adriamycin, and doxorubicin treatment by targeting *BCL2L-2*, *BCL-W,* and *RAF-1* genes, respectively [23, 43–45]. Here, we determined that transgenic expression of PHB1 neutralizes the anti-proliferative effect of miR-195, establishing PHB1 as relevant target gene. The differences observed between the UACC-62 and SK-MEL-5 cell lines can be a result of genetic heterogeneity [25].

## Conclusion

In summary, our results established miR-195-PHB1 as important regulatory node. Lacking of miR-195 expression in melanoma patients seems to be one of the main mechanisms of PHB1 accumulation in melanomas which could decrease the efficacy of chemotherapy and even target therapies like vemurafenib used in melanoma patients harboring BRAF V600E mutation. Evaluation of miR-195 and PHB1 levels could help a better selection and follow-up of patients for melanoma treatment.

## Additional files

Additional file 1: Table S1. Sources of cell lines used at this study. (DOCX 19 kb)

Additional file 2: Figure S1. MicroRNA-195 modulates PHB1 expression in melanoma cells. SK-MEL-5 melanoma cells were transfected with either miR-control/mir-195. miR-195 mimics transfection resulting in a reduction of PHB1 (*P* ≤ 0.01) (**a**) mRNA and (**b**) protein levels compared to miR-control. For RT-qPCR experiments, ACT-B mRNA was used as an endogenous control and the data were analyzed using the 2 ^(−∆∆Ct)^ method; for immunoblotting ACT-B was also used as loading control. Protein quantification (fold-change based on the control) is indicated above the blots. In (**c**) and (**d**), miR-195 levels 48 and 72 h after transfection, respectively. RNU48 was used as an endogenous control and the data were analyzed using the 2 ^(−∆∆Ct)^ method. **P ≤ 0.01. (PNG 74 kb)

Additional file 3: Figure S2. miRNA-195 act as anti-proliferative microRNA in melanoma cell. Proliferation assay based on nuclear counting per mm2. SK-MEL-5 melanoma cells were transfected with either miR-control or miR-195 (10 nM) and observed for five days after transfection Representative examples of at least three independent experiments are reported. (PNG 32 kb)

Additional file 4: Figure S3. UACC-62 stable cells expressing recombinant ORF-PHB1. UACC-62 melanoma cells were stably selected by G418 antibiotic. siRNA assays confirmed expression of recombinant PHB1. Endogenous PHB1 was used as positive control. Fold-change is indicated below the blots. PHB1 = Prohibitin 1; TUB = beta-tubulin, nV5-Tag = N-terminal V5 epitope tag for detection using Anti-V5 antibodies. (PNG 43 kb)

Additional file 5: Figure S4. MicroRNA-195 and drugs in SK-MEL-5 melanoma cells. (**a**-**b**) Cell viability rate was calculated based on the proliferation index ratio (%) of treated cells/not treated cells (control). Increasing doses of cisplatin (2.5, 5.0, and 10.0 μM) and temozolomide (50, 250, and 450 μM) were tested. (**c**-**d**) FlowJo Cytometry Analysis software was used for hypodiploid cell quantification after propidium iodide staining. Cells were treated with 2.5 and 5.0 μM cisplatin and 50 and 250 μM temozolomide drugs. (**e**-**f**) Apoptosis index based on caspase 3/7 activity was measured in a luminometer. All results showed that alone miR-195 exerts a effect in SK-MEL-5 melanoma cells compared to cisplatin and temozolomide treatments. All experimental data were obtained 24 h after miRNA-control/miR-195 (10 nM) transfection plus 48 h of drug exposure (total time 72 h). Statistical analysis were carried out using ANOVA with multiple comparison test and are reported as means ± SD. Representative data of at least three independent experiments are reported. NS: non-significant; *P ≤ 0.05; **** *P* ≤ 0.0001. (PNG 514 kb)

Additional file 6: Figure S5. MicroRNA 195 and PLX-4032 effects in UACC-62 melanoma cells. UACC-62 cells were transfected with either miR-control/miR-195 (25 nM). After 24 h, cells were treated with 1 or 10 μM vemurafenib (PLX-4032) for 48 hs and cell death was determined by flow cytometry after propidium iodide staining. Statistical analysis was carried out using Two-Way ANOVA followed by Bonferroni post-test and are reported by mean ± SD. Representative data of three independent experiments are reported. ***P ≤ 0.001. (JPEG 222 kb)

Additional file 7: Figure S6. Drug-induced cell death is accentuated by miR-195. This panel shows the cell cycle profile of SK-MEL-5 melanoma cells transfected with either miRNA-control/miR-195 (10 nM) (24 h) and treated with cisplatin (CIS-2.5 and 5 μM) or temozolomide (TMZ-50 and 250 μM) for 48 h (total time 72 h). The percentage of the cell population distributed in each cell cycle phase is indicated: G0/G1 = blue, S = green, and G2/M = pink. (**a**)-MicroRNA-195 alone increased cell death (cells accumulated at sub G0/G1). (**b**-**e**) Treatment with drugs induces mainly arrest of SK-MEL-5 cells in G2/M whereas the cytotoxic effects of cisplatin and temozolomide were higher when combined with miR-195 transfection, inducing cell death (sub G0/G1 cells population). Cell cycle distribution of propidium iodide (PI)-labeled cells was analyzed using FlowJo Cytometric software. Representative examples of at least three independent experiments are reported. (JPEG 314 kb)

Additional file 8: Figure S7. PHB1 protects UACC-62 melanoma cells of chemotherapy induced cell-death. UACC-62 melanoma cells stably expressing either pcDNA3.1-EV or ORF-PHB1 were treated with cisplatin (cis; 5 or 10 μM) or temozolomide (tmz; 50 or 250 μM) for 48 h. The percentage of viable cells (**a**) and annexin V positive/PI negative cells (**b**) were determined using Annexin V Conjugates for Apoptosis Detection kit for flow cytometry (Life Technologies). Statistical analysis was carried out using Two-Way ANOVA followed by Bonferroni post-test and are reported by mean ± SD. Representative data of three independent experiments are reported. ***P ≤ 0.001. (JPEG 62 kb)

## Abbreviations

ACT-B: actin beta
CIS: cisplatin
FBS: Fetal bovine serum
HRP: Horseradish peroxidase
IgG: immunoglobulin G
mimic: mimicking precursor of miR-195 that is double-stranded synthetic RNA oligonucleotide
miRNA: microRNA
mRNA-Seq: RNA sequencing
NGM: human melanocytes
ORF: open reading frame
PHB1: Prohibitin 1
PI: propidium iodide
RT-qPCR: quantitative real time polymerase chain reaction
SDS-PAGE: stands for sodium dodecyl sulfate - polyacrylamide gel electrophoresis
siRNA: small interfering RNA
TBS: tris-buffered saline
TCGA: The Cancer Genome Atlas
TMZ: temozolomide
TUB: tubulin
