# Supplementary material for: MicroRNA-195 acts as an anti-proliferative miRNA in human melanoma cells by targeting Prohibitin 1
Source: BMC Cancer. 2017 Nov 10;17:750. doi: 10.1186/s12885-017-3721-7 (PMC5681823; doi:10.1186/s12885-017-3721-7)
Supplement: Supplementary file 1 — Sources of cell lines used at this study. (DOCX 19 kb) [file 12885_2017_3721_MOESM1_ESM.docx]

**Additional File 1. Table 1.** Sources of cell lines used at this study.

| **Cell line** | **Supplier** | **Catalog Number** | **Reference/Cell establishment** |
| --- | --- | --- | --- |
| HaCat | CLS | 300493/p800_HaCaT | [1] |
| HeLa | ATCC | CCL-2 | [2] |
| MZ2Mel | NA | NA | [3][4] |
| NGM | BCRJ | 0190 | NA |
| SK-MEL-5 | ATCC | HTB-70 | [5] |
| SK-MEL-19 | Lonza | 1332 | [5] |
| SK-MEL-37 | NA | NA | [5] |
| SK-MEL-147 | Lonza | 1338 | [6] |
| UACC-62 | Genentech | 586090 | [7] |
| WM35 | ATCC | CRL-2806 | [8] |
| WM793B | ATCC | CRL-2807 | [8] |
| WM1366 | Coriell | WC00078 | [9] |
| WM1552C | ATCC | CRL-2808 | [8] |
| WM1617 | Coriell | WC00076 | [10] |

NA: Not Applicable; ATCC: The Global Bioresource Center; CLS: Cell Line Service; BCRJ: Banco de Células do Rio de Janeiro.

**References**

1. Boukamp P, Petrussevska RT, Breitkreutz D, Hornung J, Markham A, Fusenig NE. Normal keratinization in a spontaneously immortalized aneuploid human keratinocyte cell line. J. Cell Biol. 1988;106:761–71.

2. Gey G.O., Coffman W.D. KMT. Tissue culture studies of the proliferative capacity of cervical carcinoma and normal epithelium. Cancer Res. 1952;12:264–5.

3. Hérin M, Lemoine C, Weynants P, Vessière F, Van Pel A, Knuth A, et al. Production of stable cytolytic T-cell clones directed against autologous human melanoma. Int. J. cancer. 1987;39:390–6.

4. Iorio F, Knijnenburg TA, Vis DJ, Bignell GR, Menden MP, Schubert M, et al. A Landscape of Pharmacogenomic Interactions in Cancer. Cell. 2016;166:740–54.

5. Carey TE, Takahashi T, Resnick LA, Oettgen HF, Old LJ. Cell surface antigens of human malignant melanoma: mixed hemadsorption assays for humoral immunity to cultured autologous melanoma cells. Proc. Natl. Acad. Sci. 1976;73:3278–82.

6. Houghton AN. Detection of cell surface and intracellular antigens by human monoclonal antibodies. Hybrid cell lines derived from lymphocytes of patients with malignant melanoma. J. Exp. Med. 1983;158:53–65.

7. Ikediobi ON, Davies H, Bignell G, Edkins S, Stevens C, O’Meara S, et al. Mutation analysis of 24 known cancer genes in the NCI-60 cell line set. Mol. Cancer Ther. 2006;5:2606–12.

8. Cornil I, Theodorescu D, Man S, Herlyn M, Jambrosic J, Kerbel RS. Fibroblast cell interactions with human melanoma cells affect tumor cell growth as a function of tumor progression. Proc. Natl. Acad. Sci. 1991;88:6028–32.

9. Jönsson G, Dahl C, Staaf J, Sandberg T, Bendahl P-O, Ringnér M, et al. Genomic profiling of malignant melanoma using tiling-resolution arrayCGH. Oncogene. 2007;26:4738–48.

10. Estrada-Bernal A, Gatlin JC, Sunpaweravong S, Pfenninger KH. Dynamic adhesions and MARCKS in melanoma cells. J. Cell Sci. 2009;122:2300–10.
