# Supplementary figures and images for: MicroRNA-195 acts as an anti-proliferative miRNA in human melanoma cells by targeting Prohibitin 1
Source: BMC Cancer. 2017 Nov 10;17:750. doi: 10.1186/s12885-017-3721-7 (PMC5681823; doi:10.1186/s12885-017-3721-7)

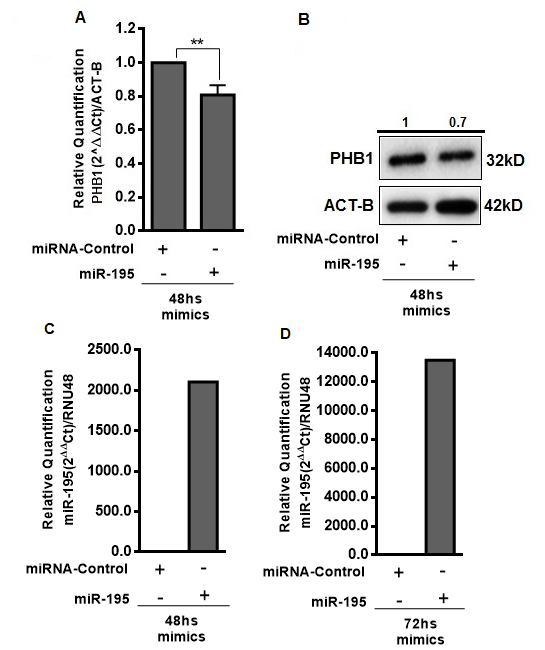

Supplement: Supplementary file 2 — MicroRNA-195 modulates PHB1 expression in melanoma cells. SK-MEL-5 melanoma cells were transfected with either miR-control/mir-195. miR-195 mimics transfection resulting in a reduction of PHB1 (P ≤ 0.01) (a) mRNA and (b) protein levels compared to miR-control. For RT-qPCR experiments, ACT-B mRNA was used as an endogenous control and the data were analyzed using the 2 (−∆∆Ct) method; for immunoblotting ACT-B was also used as loading control. Protein quantification (fold-change based on the control) is indicated above the blots. In (c) and (d), miR-195 levels 48 and 72 h after transfection, respectively. RNU48 was used as an endogenous control and the data were analyzed using the 2 (−∆∆Ct) method. **P ≤ 0.01. (PNG 74 kb) [file 12885_2017_3721_MOESM2_ESM.png]

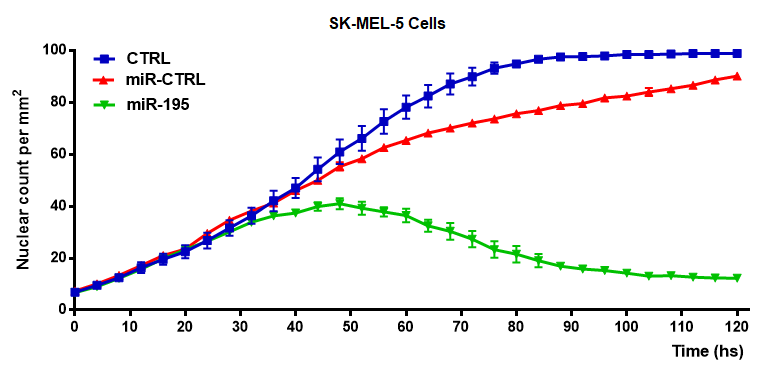

Supplement: Supplementary file 3 — miRNA-195 act as anti-proliferative microRNA in melanoma cell. Proliferation assay based on nuclear counting per mm2. SK-MEL-5 melanoma cells were transfected with either miR-control or miR-195 (10 nM) and observed for five days after transfection Representative examples of at least three independent experiments are reported. (PNG 32 kb) [file 12885_2017_3721_MOESM3_ESM.png]

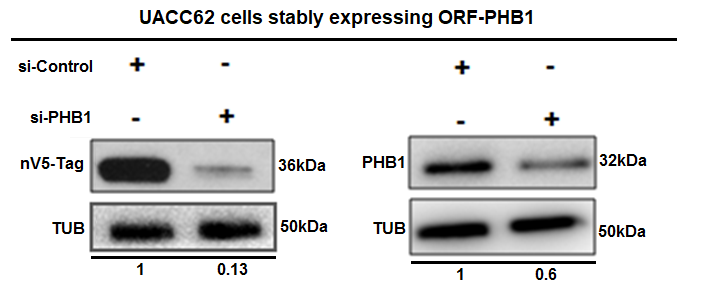

Supplement: Supplementary file 4 — UACC-62 stable cells expressing recombinant ORF-PHB1. UACC-62 melanoma cells were stably selected by G418 antibiotic. siRNA assays confirmed expression of recombinant PHB1. Endogenous PHB1 was used as positive control. Fold-change is indicated below the blots. PHB1 = Prohibitin 1; TUB = beta-tubulin, nV5-Tag = N-terminal V5 epitope tag for detection using Anti-V5 antibodies. (PNG 43 kb) [file 12885_2017_3721_MOESM4_ESM.png]

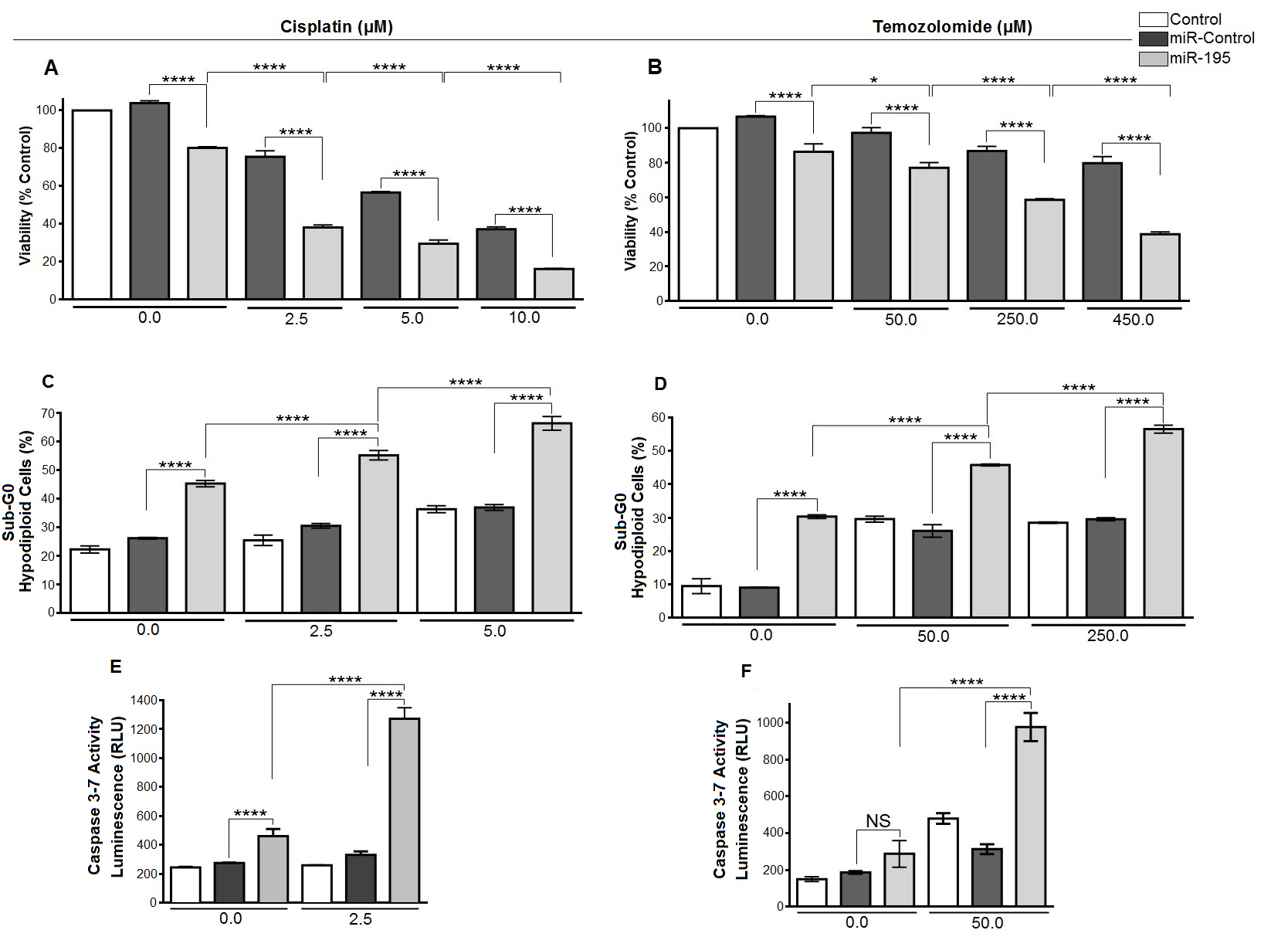

Supplement: Supplementary file 5 — MicroRNA-195 and drugs in SK-MEL-5 melanoma cells. (a-b) Cell viability rate was calculated based on the proliferation index ratio (%) of treated cells/not treated cells (control). Increasing doses of cisplatin (2.5, 5.0, and 10.0 μM) and temozolomide (50, 250, and 450 μM) were tested. (c-d) FlowJo Cytometry Analysis software was used for hypodiploid cell quantification after propidium iodide staining. Cells were treated with 2.5 and 5.0 μM cisplatin and 50 and 250 μM temozolomide drugs. (e-f) Apoptosis index based on caspase 3/7 activity was measured in a luminometer. All results showed that alone miR-195 exerts a effect in SK-MEL-5 melanoma cells compared to cisplatin and temozolomide treatments. All experimental data were obtained 24 h after miRNA-control/miR-195 (10 nM) transfection plus 48 h of drug exposure (total time 72 h). Statistical analysis were carried out using ANOVA with multiple comparison test and are reported as means ± SD. Representative data of at least three independent experiments are reported. NS: non-significant; *P ≤ 0.05; **** P ≤ 0.0001. (PNG 514 kb) [file 12885_2017_3721_MOESM5_ESM.png]

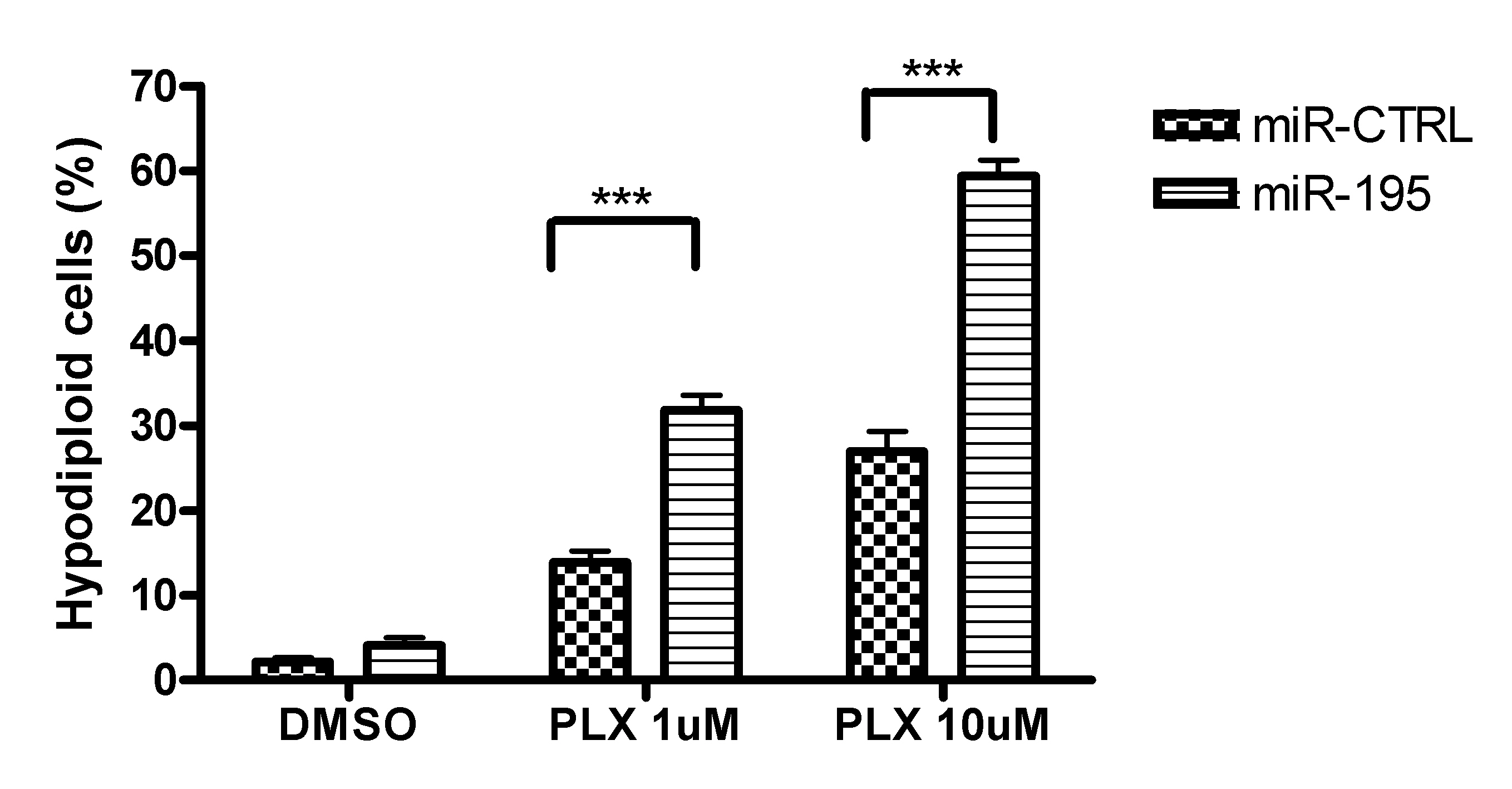

Supplement: Supplementary file 6 — MicroRNA 195 and PLX-4032 effects in UACC-62 melanoma cells. UACC-62 cells were transfected with either miR-control/miR-195 (25 nM). After 24 h, cells were treated with 1 or 10 μM vemurafenib (PLX-4032) for 48 hs and cell death was determined by flow cytometry after propidium iodide staining. Statistical analysis was carried out using Two-Way ANOVA followed by Bonferroni post-test and are reported by mean ± SD. Representative data of three independent experiments are reported. ***P ≤ 0.001. (JPEG 222 kb) [file 12885_2017_3721_MOESM6_ESM.jpg]

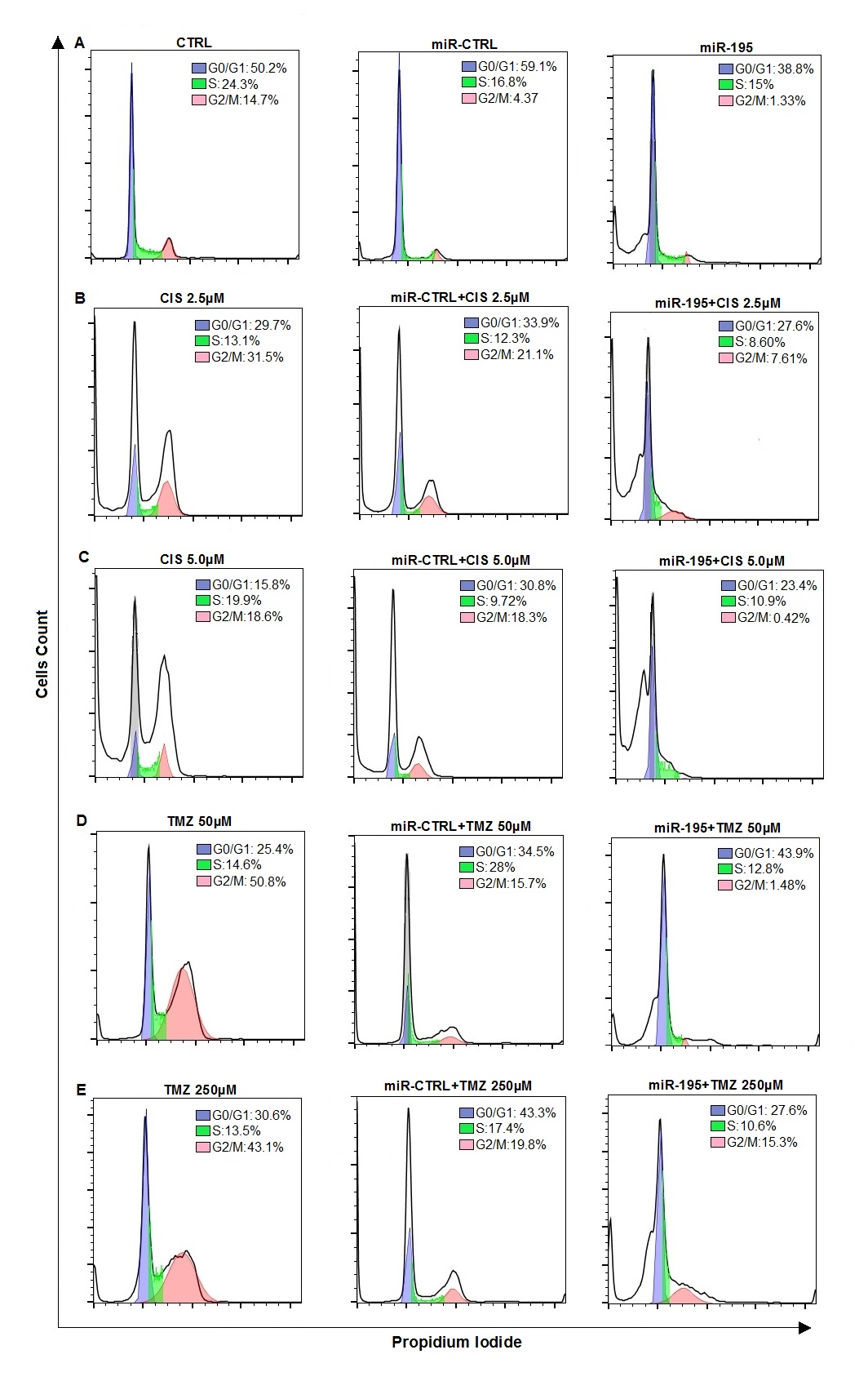

Supplement: Supplementary file 7 — Drug-induced cell death is accentuated by miR-195. This panel shows the cell cycle profile of SK-MEL-5 melanoma cells transfected with either miRNA-control/miR-195 (10 nM) (24 h) and treated with cisplatin (CIS-2.5 and 5 μM) or temozolomide (TMZ-50 and 250 μM) for 48 h (total time 72 h). The percentage of the cell population distributed in each cell cycle phase is indicated: G0/G1 = blue, S = green, and G2/M = pink. (a)-MicroRNA-195 alone increased cell death (cells accumulated at sub G0/G1). (b-e) Treatment with drugs induces mainly arrest of SK-MEL-5 cells in G2/M whereas the cytotoxic effects of cisplatin and temozolomide were higher when combined with miR-195 transfection, inducing cell death (sub G0/G1 cells population). Cell cycle distribution of propidium iodide (PI)-labeled cells was analyzed using FlowJo Cytometric software. Representative examples of at least three independent experiments are reported. (JPEG 314 kb) [file 12885_2017_3721_MOESM7_ESM.jpg]

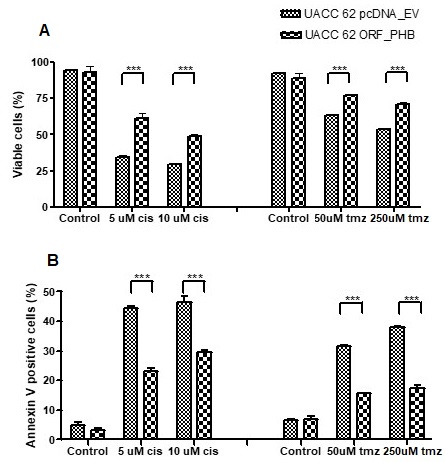

Supplement: Supplementary file 8 — PHB1 protects UACC-62 melanoma cells of chemotherapy induced cell-death. UACC-62 melanoma cells stably expressing either pcDNA3.1-EV or ORF-PHB1 were treated with cisplatin (cis; 5 or 10 μM) or temozolomide (tmz; 50 or 250 μM) for 48 h. The percentage of viable cells (a) and annexin V positive/PI negative cells (b) were determined using Annexin V Conjugates for Apoptosis Detection kit for flow cytometry (Life Technologies). Statistical analysis was carried out using Two-Way ANOVA followed by Bonferroni post-test and are reported by mean ± SD. Representative data of three independent experiments are reported. ***P ≤ 0.001. (JPEG 62 kb) [file 12885_2017_3721_MOESM8_ESM.jpg]
